# Poor prognosis indicators of type-2 diabetic COVID-19 patients

**DOI:** 10.1590/1414-431X2022e11819

**Published:** 2022-06-22

**Authors:** R. Gorjão, S.M. Hirabara, L.N. Masi, T.D.A. Serdan, R.B. Gritte, E. Hatanaka, T. Souza-Siqueira, A.C. Pithon-Curi, T.M. de Lima, T.C. Pithon-Curi, J.F.M. Marchini, M.C.C. Machado, H.P. Souza, R. Curi

**Affiliations:** 1 Programa de Pós-Graduação Interdisciplinar em Ciências da Saúde, Universidade Cruzeiro do Sul, São Paulo, SP, Brasil; 2 Laboratório de Emergências Clínicas (LIM-51), Faculdade de Medicina, Universidade de São Paulo, São Paulo, SP, Brasil; 3 Setor Bioindustrial, Instituto Butantan, São Paulo, SP, Brasil; 4 Department of Molecular Pathobiology, New York University, New York, NY, USA

**Keywords:** SARS-CoV-2, C-reactive protein, Lactate, Urea, D-dimer, NEWS

## Abstract

Diabetes is associated with a worse prognosis and a high risk of morbidity and mortality in COVID-19 patients. We aimed to evaluate the main factors involved in the poor prognosis in diabetic patients. A total of 984 patients diagnosed with COVID-19 admitted to the hospital were included in this study. Patients were first divided into type-2 diabetic (DM+) and non-diabetic (DM–) groups. The participants were analyzed based on the National Early Warning Score (NEWS) and on the Quick-Sequential Organ Failure Assessment (qSOFA) to find the best prognostic risk score for our study. The DM+ and DM– groups were divided into non-severe and severe groups. Comparative and correlative analyses were used to identify the physiological parameters that could be employed for creating a potential risk indicator for DM+ COVID-19 patients. We found a poorer prognosis for the DM+ COVID-19 patients with a higher ICU admission rate, mechanical ventilation rate, vasopressor use, dialysis, and longer treatment times compared with the DM– group. DM+ COVID-19 patients had increased plasma glucose, lactate, age, urea, NEWS, and D-dimer levels, herein referred to as the GLAUND set, and worse prognosis and outcomes when compared with infected DM– patients. The NEWS score was a better indicator for assessing COVID-19 severity in diabetic patients than the q-SOFA score. In conclusion, diabetic COVID-19 patients should be assessed with the NEWS score and GLAUND set for determining their prognosis COVID-19 prognosis.

## Introduction

The severe acute respiratory syndrome coronavirus-2 (SARS-CoV-2) causes the coronavirus disease-2019 (COVID-19). This disease emerged in China (Hubei province of Wuhan) in late December 2019 and spread rapidly worldwide. In late February 2020, the disease was first detected in São Paulo, SP, Brazil, and spread throughout the country after a few weeks (1,2). Brazil became the pandemic center in Latin America in May 2020 (3-7). The country had more than 29.1 million confirmed cases and 652,829 deaths (2.24% mortality rate) on March 10, 2022. At that time, the United States and India were the only two countries with more confirmed cases than Brazil, and the former had the most cases (more than 78.6 million people) and deaths (954,913) in the world. As of March 10, 2022, more than 450.2 million confirmed cases have been reported worldwide, and 6,019,085 people (1.33% case-fatality rate) have died from COVID-19.

The prognosis of COVID-19 and its progression varies drastically depending on the patient’s characteristics such as their health status and comorbidities. Recently, the characteristics highly associated with severe COVID-19 and high mortality rate were reported, and included age, elevated plasma C-reactive protein (CRP), D-dimer levels, and troponin T, decreased plasma albumin levels, increased body temperature, raised SOFA score, elevated pulmonary angiotensin-converting enzyme 2 (ACE2) expression, reduced plasma lymphocyte amount, extended ground glass opacities on chest computed tomography, and a high prevalence of comorbidities, including diabetes mellitus (4-7). We point out that diabetes is the most common comorbidity associated with severe cases of COVID-19 (8). Several factors are associated with poor prognosis of COVID-19 in type-2 diabetic patients, including advanced age, proinflammatory condition, hypercoagulation process, hyperglycemia, and related comorbidities (9,10).

Diabetic patients exhibit a chronic low-grade inflammatory state associated with dysregulation of the immune system (11). This condition induces the activation of adaptive and innate immune cells in white adipose tissue and is accompanied by elevated levels of pro-inflammatory factors including interleukin (IL)-6, tumor necrosis factor alpha (TNF-α), and c-reactive protein (CRP) (12,13). Indeed, alterations in the immune system and inflammatory state are associated with increased risk of viral infections from H1N1, SARS-CoV, and Middle East respiratory syndrome coronavirus (MERS-CoV) (14-16).

Several studies have reported that diabetic COVID-19 patients have a poorer prognosis and a high risk of morbidity and mortality (17-20). These features are due to the preexisting low-grade inflammation and immune function dysregulation in diabetic patients mentioned above, which contribute to the cytokine storm that develops by mechanisms not yet fully understood in the later stages of coronavirus infections (21). COVID-19 patients exhibit elevated total blood leukocytes and neutrophils, decreased lymphocyte counts, increased neutrophil/lymphocyte ratios (NLR), and high hepatic fibrosis-4 index (14,22-24). Impaired fasting glucose and poor control of hyperglycemia are also correlated to the severity and mortality in type-2 diabetic COVID-19 patients (9,25). On the one hand, when type-2 diabetic patients have a history of metformin treatment before the COVID-19 diagnosis, there is a decrease in the risk of mortality (26). Another important point identified is that statin use was associated with reduced mortality from COVID-19 in patients with diabetes mellitus (27). On the other hand, Yu et al. (28) unexpectedly observed that insulin treatment for COVID-19 patients with type-2 diabetes mellitus increased mortality (27.2 *vs* 3.5%). However, the main reasons for the poorer COVID-19 prognosis in diabetic patients are still under investigation. Thus, the present study aimed to evaluate the main clinical factors involved in the prognosis of COVID-19 in type-2 diabetic patients.

## Material and Methods

The Institutional Ethics Committee of the University of São Paulo School of Medicine (Brazil) approved the present study under the number 3.990.817 (CAAE: 30417520.0.0000.0068). Since we adhered to the Strengthening the Reporting of Observational Studies in Epidemiology (STROBE) guidelines, the need for written informed consent was not required. In the present study, we evaluated several physiological parameters in type-2 diabetic and non-diabetic COVID-19 patients.

### Participants

Participants were eligible for this study if diagnosed with COVID-19, older than 18 years and admitted to the Emergency Department of Hospital das Clínicas at the University of São Paulo School of Medicine, located in São Paulo, Brazil, from February to May 2020. In this period, we enrolled 984 individuals. We followed World Health Organization (WHO) recommendations for diagnosing COVID-19 infection. Briefly, COVID-19 was diagnosed with reverse transcription-polymerase chain reaction (RT-PCR) of nasopharyngeal or tracheal exudate. Patients were first divided into two groups: type-2 diabetic (DM+) and non-diabetic (DM–). Type-1 diabetic patients were excluded from this study. Initially, the participants were analyzed based on the National Early Warning Score (NEWS) and on the Quick-Sequential Organ Failure Assessment (qSOFA) to find the best risk prognostic indicator for our study. After that, we divided the DM+ and DM– groups into non-severe and severe groups and analyzed several laboratory parameters.

### Laboratory data

The medical history, symptoms, laboratory findings, and treatment were obtained through electronic medical records. Laboratory data were obtained using clinical assays performed according to the hospital’s standard methods. The following blood data were evaluated: CRP levels, D-dimer levels, complete blood count, white blood cell counts (total leukocytes, neutrophils, lymphocytes, monocytes, eosinophils, basophils, and platelets), red cell distribution [coefficient of variation (CV) and standard deviation (SD)], metabolites (glucose, lactate, albumin, urea, and creatinine), markers of tissue damage [lactate dehydrogenase (DHL), creatine phosphokinase (CPK), aspartate aminotransferase (AST), alanine aminotransferase (ALT), creatine kinase-MB (CKMB), troponin T, electrolytes (sodium, potassium, sodium/potassium ratio, phosphate, magnesium, calcium), bilirubin [total (BT), direct (BD), and indirect (BI)]], activated partial thromboplastin time (TTPA), parameters related to red blood cells [hemoglobin, hematocrit, mean corpuscular volume (MCV)], mean corpuscular hemoglobin (MCH), mean corpuscular hemoglobin concentration (MCHC), red cell distribution width (RDW), and parameters associated with blood gas analysis [potential hydrogen (pH), bicarbonate (HCO_3_-), sulfur dioxide (SO_2_), oxygen partial pressure (pO_2_), oxyhemoglobin fraction (FO_2_Hb), carboxyhemoglobin fraction (FCOHb), methemoglobin fraction (FMetHb), and carbon dioxide partial pressure (pCO_2_)]. We also calculated several ratios associated with the worst COVID-19 prognosis, including neutrophil/lymphocyte ratio (NLR), derived-NLR (d-NLR), lymphocyte/neutrophil ratio (LNR), d-LNR, platelet/lymphocyte ratio (PLR), RDW-CV/lymphocyte ratio (RLR-CV), and RDW-SD/lymphocyte ratio (RLR-SD).

### Severity definition of COVID-19

We used two risk factors, NEWS and qSOFA, as previously proposed for evaluation of COVID-19 severity (4,29), and evaluated their correlation with some well-known parameters that are increased during the severe stage of COVID-19, including the total amount of circulating leukocytes and neutrophils, and plasma levels of CRP and D-dimer.

### NEWS

We determined the severity of the condition using the NEWS that was created at the Royal College of Physicians in 2012. The score is based on six physiological parameters: respiration rate, oxygen saturation, systolic blood pressure, pulse rate, level of consciousness or new confusion, and temperature (30). The NEWS varies between 0 to 19, and scores of ≤4 correspond to low severity, 5-6 intermediate severity, and ≥7 high severity.

### q-SOFA

The Quick-Sequential Organ Failure Assessment (qSOFA) score helps identify patients with infection and a poor outcome after leaving the intensive care unit (ICU). The qSOFA considers one point for low blood pressure [systolic blood pressure (SBP) ≤100 mmHg], one point for high respiratory rate (≥22 breaths per min), and one point for altered level of consciousness (Glasgow coma scale <15). The qSOFA scores can range from 0 to 3 points. A high risk of death or prolonged ICU stay is associated with qSOFA scores of ≥2. The Third International Consensus Definitions for Sepsis supported the use of qSOFA to identify septic patients outside the ICU (31).

### Patient outcomes

The outcomes of 588 participants were assessed: 185 DM– non-severe, 184 DM– severe, 95 DM+ non-severe, and 124 DM+ severe. This analysis was based on the percentage of patients requiring mechanical ventilation, vasopressor drugs and/or dialysis in the ICU, and the duration (in days) of these treatments. We also determined the mortality rate of the four groups. As discussed in the text, the classification of non-severe and severe was based on the NEWS assessment.

### Prognosis of diabetic COVID-19 patients using the GLAUND set

Following the comparative and correlation analyses, we identified and selected six physiological measures to propose a prognostic indicator for diabetic patients with COVID-19, referred to as the GLAUND set. This acronym (GLAUND) stands for Glucose, Lactate, Age, Urea, NEWS, and D-dimer indicators. Physiological parameters with statistical significance (P<0.05) or a tendency of significance (P<0.20) were identified. Briefly, the GLAUND set is calculated by dividing the values of each parameter by the reference values and then summing the resulting values. The GLAUND set = (glycemia/100 mg/dL) + (lactate/15 mg/dL) + (age/60 years) + (urea/50 mg/dL) + (NEWS/4) + (D-dimer/600 ng/dL).

### Statistical analysis

Quantitative variables are reported as means±SE for all biochemical parameters. The Shapiro-Wilk normality test was used to determine the normality of the data distribution. Two-way analysis of variance (two-way ANOVA) was used to compare the severity of COVID19 infection and the presence or not of diabetes. The Student's *t*-test was used to compare two groups when a tendency of statistical difference (P value between 0.05 and 0.20) was observed in the two-way ANOVA. Correlation coefficients were then calculated between different laboratory parameters and NEWS score, using Pearson's correlation. The intercept value was analyzed because it indicates the Y value when X=0. In other words, the intercept shows the parameter value at very low risk for severe disease (when the risk factor NEWS is equal to zero). Chi-squared test was used to compare non-severe and severe groups, and DM+ and DM– groups. Statistical analysis using the GLAUND set was performed by two-way ANOVA to compare the severity of COVID19 infection in patients with and without type-2 diabetes. A P-value of ≤0.05 was considered as the criterion for statistical significance. All statistical analyses were performed using GraphPad Prism version 7.0 (GraphPad Software Inc., USA).

## Results

### Comparison of NEWS and qSOFA for the evaluation of COVID-19 severity

Because there was no difference between the low and intermediate severity groups according to the NEWS scale (data not shown), we pooled these two groups into the non-severe group.

**Figure 1:**
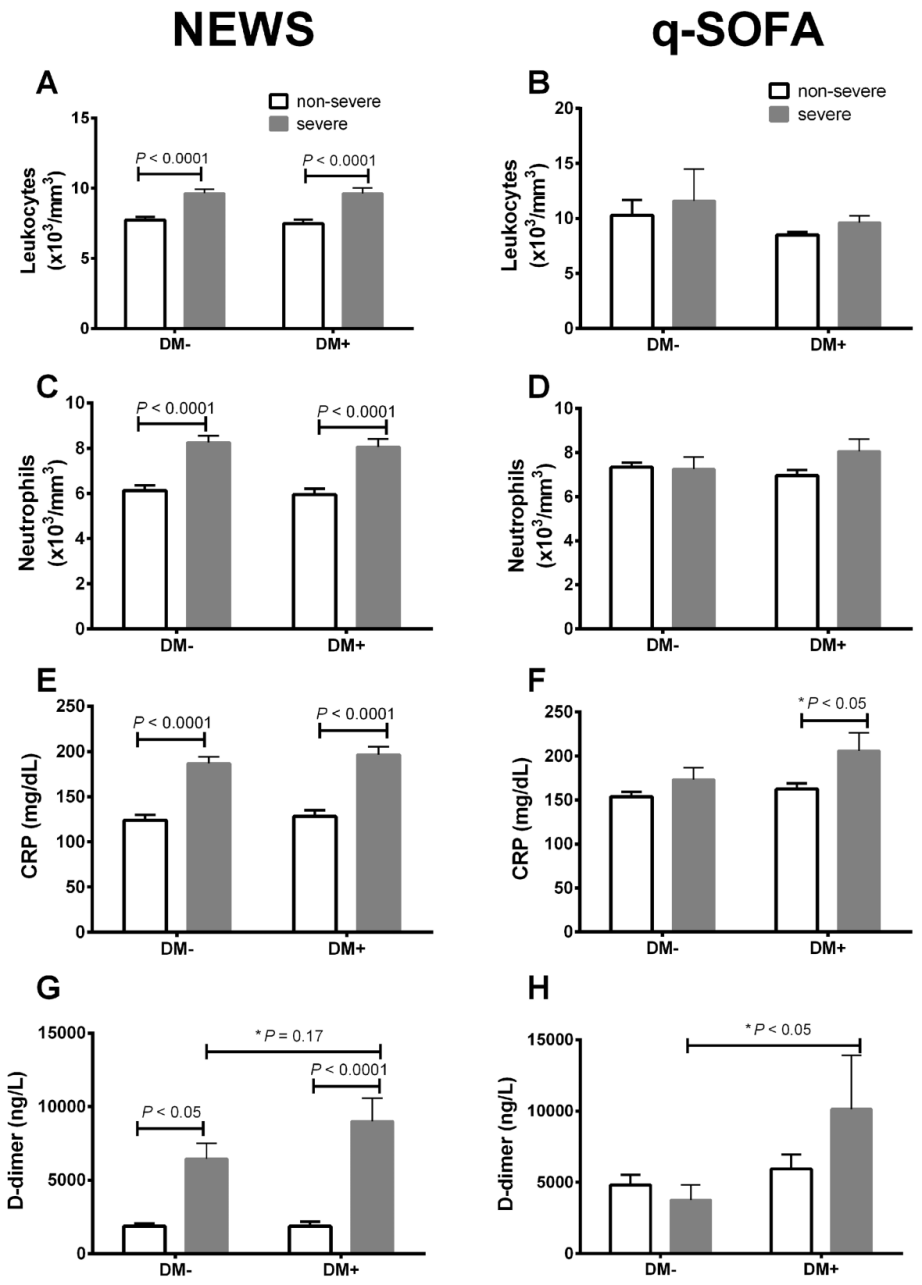
Comparative analysis between the National Early Warning Score (NEWS) and the Quick-Sequential Organ Failure Assessment (qSOFA) in non-diabetic (DM–) and type-2 diabetic (DM+) patients. Total amount of leukocytes (**A** and **B**) and neutrophils (**C** and **D**) and plasma levels of C-reactive protein (CRP) (**E** and **F**) and D-dimer (**G** and **H**). Data are reported as means±SE. P<0.05, ANOVA and *t*-test (P value with an asterisk).

The NEWS score was a better risk indicator than qSOFA, considering parameters that are known to increase in severe COVID-19, including levels of total leukocytes, neutrophils, plasma CRP, and D-dimer levels in the severe groups compared to the non-severe groups, regardless of the diabetic state (Figure 1). The q-SOFA scale failed to detect any significant differences in all these parameters between the non-severe and severe groups, in non-diabetic and diabetic patients (Figure 1). The qSOFA scale was able to detect a small difference in CRP (DM+ non-severe *vs* DM+ severe) and D-dimer (DM– severe *vs* DM+ severe) by the Student's *t-*test (Figure 1).

### COVID-19 severity in non-diabetic and diabetic patients

Due to the better ability of the NEWS scale to discriminate COVID-19 severity in DM– and DM+ patients, we opted to use this scale for classifying these patients in the subsequent analyses. Based on this classification approach, a summary of the significantly different laboratory data between the groups is presented in Supplementary Tables S1 and S2.

**Figure 2:**
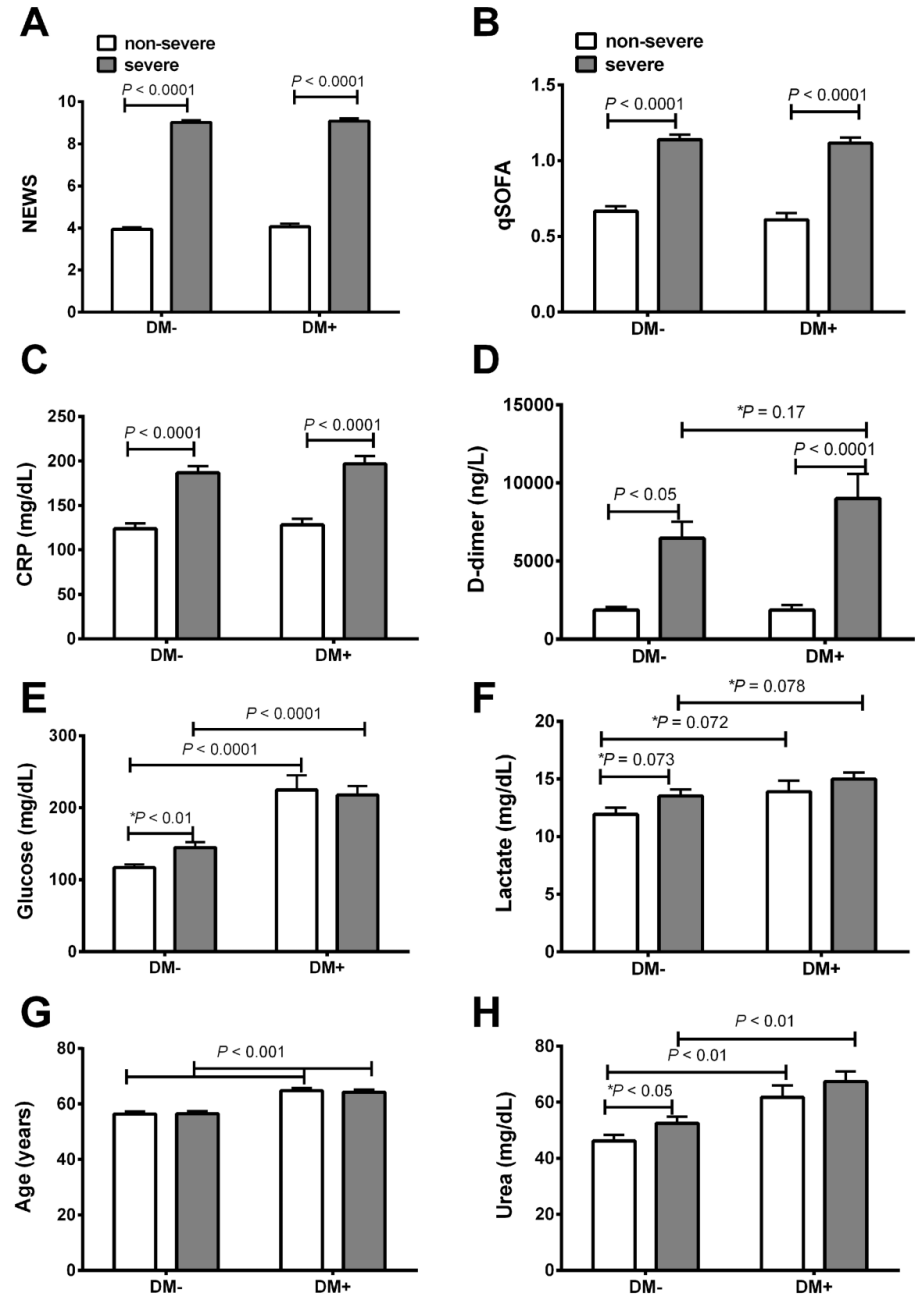
Risk scores and plasma biochemical parameters in non-diabetic (DM–) and type-2 diabetic (DM+) patients with COVID-19 classified as non-severe and severe according to the National Early Warning Score (NEWS). NEWS (**A**), Quick-Sequential Organ Failure Assessment (qSOFA) (**B**), C-reactive protein (CRP) (**C**), D-dimer (**D**), plasma glucose (**E**), lactate (**F**), age (**G**), and urea (**H**) in type 2 diabetic and non-diabetic patients. Data are reported as means±SE. P<0.05, ANOVA and *t*-test (P value with an asterisk).

Upon closer examination of the laboratory values, COVID-19 severity was associated with higher NEWS score (P<0.0001), qSOFA score (P<0.0001), plasma CRP (P<0.0001), and D-dimer (P<0.05) in both DM– and DM+ groups. Plasma glucose (P<0.01) and urea levels were elevated (P<0.001) in both non-severe and severe cases of DM+ patients compared to their respective cases in DM– patients (Figure 2). Additionally, a tendency for statistical significance was observed for plasma lactate concentration in DM+ compared to the DM– group (Figure 2F). The age of the DM+ patients was slightly higher than the DM– patients (Figure 2G).

**Figure 3:**
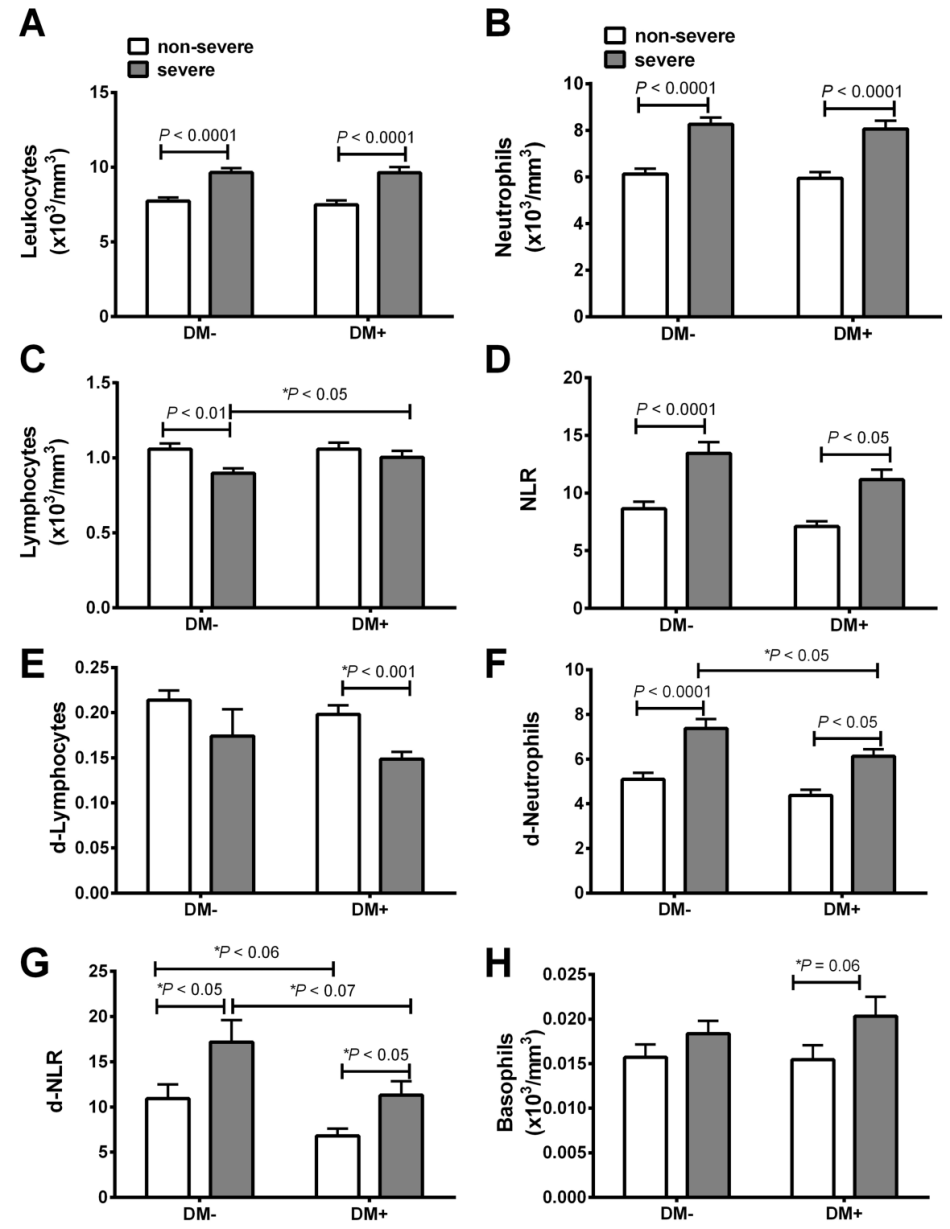
Plasma cellular parameters in non-diabetic (DM–) and type-2 diabetic (DM+) patients with COVID-19 classified as non-severe and severe according to the National Early Warning Score (NEWS). Total leukocytes (**A**), neutrophils (**B**), lymphocytes (**C**), neutrophil to lymphocyte ratio (NLR) (**D**), d-lymphocytes (**E**), d-neutrophils (**F**), d-NLR (**G**), and basophils (**H**). Data are reported as means±SE. P<0.05, ANOVA and *t*-test (P value with an asterisk).

We observed increased total leukocytes (P<0.0001), neutrophilia (P<0.001), and d-neutrophils in severe patients compared with non-severe patients (Figure 3) in both the DM– and DM+ groups. The NLR was also higher in severe DM– (P<0.001) and DM+ (P<0.05) cases compared to the respective non-severe groups (Figure 3). A significantly lower total lymphocyte count was observed only in the DM– severe group compared to the DM– non-severe group (Figure 3C). By Student’s *t*-test, we observed a lower d-lymphocyte level and a tendency of higher basophil level in DM+ severe compared to DM+ non-severe (Figures 3E and 3H, respectively).

The literature has reports of patients with severe COVID-19 exhibiting liver lesions. Moreover, this condition is associated with elevated serum ALT and AST activities. Herein, we observed increased ALT (P<0.05) and AST (P<0.05) activities in severe DM– patients when compared to non-severe DM– cases, but these differences were not present in the DM+ group. Interestingly, both DM– and DM+ patients with severe COVID-19 had increased blood DHL activity (P<0.001) compared with non-severe patients. Additionally, severe DM– patients presented low partial gas pressures of dissolved carbon dioxide (pCO_2_) in the blood compared to non-severe cases, but this difference was not detected in DM+ patients.

The activity of plasma CKMB was higher in severe DM– patients (P<0.05) compared with non-severe cases; however, despite a substantial increase in severe DM+ patients, the difference was not significant. Calcium levels in severe DM– patients were significantly decreased (P<0.05) compared with non-severe patients. Both DM– and DM+ patients with severe COVID-19 presented elevated blood magnesium levels (P<0.05 and P<0.001, respectively) compared with non-severe cases. Additionally, blood magnesium levels in non-severe DM– patients were higher than in the non-severe DM+ group. Blood phosphate levels in severe DM– and DM+ patients were significantly increased (P<0.01 and P<0.05, respectively) compared to the non-severe groups.

**Figure 4:**
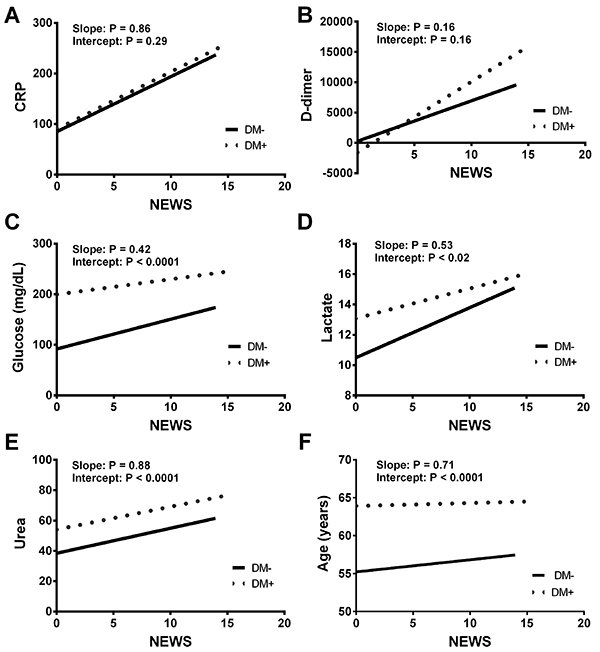
Correlation analysis between the National Early Warning Score (NEWS) and plasma C-reactive protein (CRP) (**A**), D-dimer (**B**), glucose (**C**), lactate (**D**), and urea (**E**) levels, and age (**F**) in type 2 diabetic (DM+) and non-diabetic (DM) COVID-19 patients.

There was a tendency for the NEWS score to be correlated with D-dimer when comparing the DM– and DM+ patients (P=0.16; Figure 2A). On the other hand, we did observe significant intercept differences between DM– and DM+ patients when NEWS was correlated with glucose (Figure 4C), lactate (Figure 4D), urea (4E), and age (Figure 4F). However, we did not observe any difference in the intercept between DM– and DM+ patients when the score was plotted against CRP levels (Figure 4A).

The following results were not significantly different between the groups (data not shown): monocytes, eosinophils, platelets, d-NLR, d-L, LNR, d-LNR, LMR, PLR, RDW-CV, RLR-CV, RLR-SD, albumin, creatinine, troponin T, ALT, Na/K, HB, BT, BI, BD, TTPA, HCM, CHCM, and blood gas analyses (SO_2_, FO_2_Hb, FCOHb, CT-CO_2_, HHB, HCO_3_, and FMETHb).

### Outcomes of COVID-19 diabetic patients

DM+ patients were more frequently admitted to the ICU than DM– patients (60.6 *vs* 51.8%, respectively; P<0.05). Additionally, there was a strong tendency for DM+ patients to require dialysis (19.3 *vs* 13.3%; P=0.053) in comparison to the non-diabetic patients. No differences were found for requirement of mechanical ventilation (40.3 *vs* 47.2%; P=0.09) and vasopressors (31.8 *vs* 38.2%; P=0.11) between non-diabetic and diabetic patients, respectively. In general, diabetic patients also presented higher mortality rate than non-diabetic patients (35.6 and 27.3%, respectively; P<0.05).

**Figure 5:**
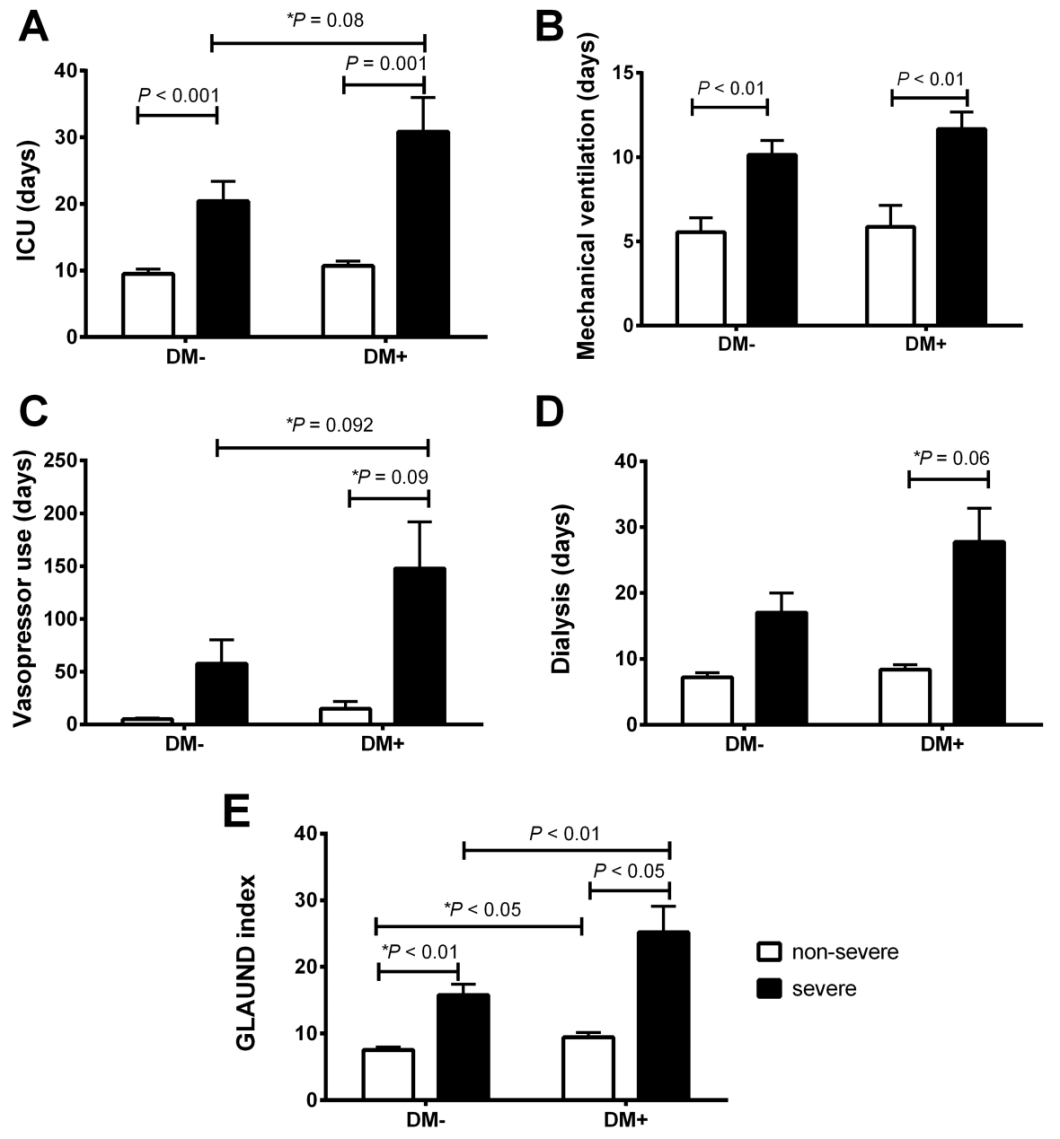
Number of days in the ICU (**A**), with mechanical ventilation (**B**), receiving vasopressor drugs (**C**), and on dialysis (**D**) of type-2 diabetic (DM+) and non-diabetic (DM–) patients classified as non-severe and severe, according to their National Early Warning Score (NEWS). The GLAUND (**E**) index scores were calculated using plasma glucose levels, plasma lactate levels, age, plasma urea levels, NEWS score, and plasma D-dimer levels. ICU: intensive care unit. Data are reported as means±SE. P<0.05, ANOVA followed by Bonferroni post hoc test and *t*-test (P value with an asterisk).

The outcomes of DM– and diabetic DM+ patients with COVID-19 classified as non-severe or severe according to the NEWS scale are displayed in Supplementary Table S1 (number of patients with different outcomes) and Figure 5 (number of days in each condition). Severe diabetic patients presented poorer prognosis as these patients required longer periods in the ICU, more mechanical ventilation, vasopressor drugs, and dialysis. It is important to observe that GLAUND differences between DM+ and DM– patients were accompanied by differences between these clinical outcomes (see below).

### Prognosis of COVID-19 in type-2 diabetic patients using the GLAUND set

Our analyses revealed six parameters that were higher in DM+ COVID-19 compared to DM– COVID-19 patients: plasma glucose levels, plasma lactate levels, age, plasma urea levels, NEWS, and plasma D-dimer levels. Therefore, the prognosis was based on significant prognostic factors found in the diabetic population studied.

As shown in Figure 5E, diabetic patients in the non-severe and severe groups presented higher GLAUND values than in the non-diabetic groups. Moreover, the GLAUND set was lower in the non-severe DM– group compared to the non-severe DM+ group (7.54±0.43 *vs* 9.44±0.72, respectively; P<0.05). Notably, a higher difference was observed when comparing the severe DM– and DM+ groups (15.7±1.65 *vs* 25.1±3.94, respectively; P<0.01).

## Discussion

A cohort study involving 660 COVID-19 patients admitted to the Central Hospital of Wuhan from January 1, 2020, to February 15, 2020, compared the risk factors between non-survivors (n=82) and survivors (n=578) (32). The authors found that advanced age, high SOFA scores, previous cerebral infarction, elevated plasma CRP levels (>0.6 mg/dL), and high lactate dehydrogenase activity (>245 U/L) increased the risk of death. Additionally, Rod et al. (4) reported that age, CRP levels, D-dimer levels, albumin levels, elevated body temperature, and SOFA scores were associated with disease progression and a poor prognosis in diabetic COVID-19 patients.

In contrast to the studies mentioned above, we did not detect a significant difference in the q-SOFA scores of DM+ and DM– COVID-19 patients. Moreover, these scores were poorly related to risk factors associated with the disease, such as CRP, D-dimer, total leukocyte, and neutrophil levels. Thus, we also evaluated disease severity using the NEWS score. We found that the NEWS score was a better indicator of COVID-19 severity in DM+ and DM– patients, and that it was also associated with CRP, D-dimer, total leukocyte, and neutrophil levels. Based on these results, we decided to use the NEWS score to classify the patients as either non-severe (low/intermediate cases) and severe.

In September 2020, Knight et al. (33) suggested that the International Severe Acute Respiratory and Emerging Infections Consortium (ISARIC) prognostic score can be applied for mortality risk stratification of COVID-19 patients. The inclusion of this assessment would be interesting in our study, but unfortunately, all data from our study were collected between February to May 2020 and, at that time, we did not collect all the parameters required for the calculation of the ISARIC score.

As mentioned above, diabetic patients present a poorer prognosis, high morbidity, and increased mortality risk when infected with SARS-CoV-2 (17-20). Accordingly, we also observed that the DM+ COVID-19 patients had a worse prognosis. For example, these patients were more likely to be admitted to the ICU and/or require mechanical ventilation, vasopressors, and dialysis than DM– patients. It should also be pointed out that DM+ COVID-19 patients also spent more time and/or received these treatments for more days than the DM– group during hospitalization.

Immune system alterations have been positively correlated with inflammatory state and COVID-19 severity (13,17,34). Increased levels of several proinflammatory cytokines and receptors, including IL-2R, IL-6, IL-8, IL-10, and TNF-α, and reduction in the peripheral total T lymphocytes, CD4+ T cells, CD8+ T cells, and NK cells have been found to be associated with high risk of mortality in COVID-19 patients with type 2 diabetes (26). We did not detect any significant difference in leukocyte levels, different leukocyte ratios, or plasma CRP levels between DM+ and DM– groups. Therefore, it does not appear that diabetes impairs the inflammatory response to viral infection. It is important to highlight that, in our study, the patients presented several comorbidities, including cardiovascular diseases (hypertension, ischemic heart disease, heart failure), respiratory diseases (chronic obstructive pulmonary disease, asthma, pneumonia), chronic inflammatory diseases (cirrhosis, lupus), and others (cancer, chronic kidney failure, and HIV). The most frequent comorbidity observed in the patients analyzed in the present study was hypertension, with 54.2% of occurrence in all participants. However, we did not find any association between any comorbidity and COVID-19 severity in diabetic patients. The occurrence of three or more comorbidities (excluding DM) was low in all groups (<10%).

It was recently reported that 29% of diabetic patients hospitalized for COVID-19 underwent tracheal intubation for assisted mechanical ventilation (34,35). Consistent with this observation, we found a strong tendency for diabetic patients to require mechanical ventilation (47.2%), vasopressor drugs (38.2%), and dialysis (19.3%). Additionally, severe COVID-19 DM+ patients also spent more days in the ICU than infected DM– patients, thus demonstrating a worse prognosis. Diabetes is also associated with hospital readmissions and poor prognosis due to other respiratory diseases, including influenza (36). Diabetic patients present increased susceptibility to develop pulmonary dysfunction (37). Diabetic animal model studies reported that alveolar capillary microangiopathy and interstitial fibrosis are involved with glycosylation of the lung tissue collagen (35,38). These alterations are associated with higher risk for lung dysfunction and need for mechanical ventilation.

The GLAUND index was determined by using comparative and correlation analyses of DM– and DM+ COVID-19 patients. Our analyses revealed six physiological parameters: plasma glucose levels, plasma lactate levels, age, plasma urea levels, NEWS, and plasma D-dimer levels. Other factors, including several biochemical and plasma parameters, did not present statistical differences or tendency for significance between DM– with DM+ patients. These parameters have been found to be increased and/or to contribute to poor prognosis of type 2 diabetic COVID-19 patients (6,9,29,39). It is important to observe that GLAUND differences between DM+ and DM– patients were accompanied by differences between these clinical outcomes. In fact, we found that DM+ individuals with non-severe and severe cases of COVID-19 present increased GLAUND compared to infected DM– patients. While the GLAUND set could be a potentially useful prognostic indicator for diabetic COVID-19 patients, we did not calculate the GLAUND set for all participants and did not validate the applicability of the GLAUND set. Studies with larger samples are required.

Despite the limitations mentioned above, three main conclusions were reached: 1) the NEWS score is a better indicator for assessing COVID-19 severity in diabetic patients than the q-SOFA score; 2) DM+ patients have a worse prognosis than DM– patients, as evidenced by an increased risk for ICU admission, mechanical ventilation, vasopressor drug requirement, dialysis, longer treatment, and higher mortality rate; and 3) the GLAUND set could be a valuable tool for determining the prognosis of type-2 diabetic COVID-19 patients.

## Supplementary Material

Click to view [pdf].
